# Development and Application of *Lactobacillus plantarum* PSCPL13 Probiotics in Olive Flounder (*Paralichthys olivaceus*) Farming

**DOI:** 10.3390/microorganisms13010061

**Published:** 2025-01-02

**Authors:** Muhammad Aleem Abbas, Hae-Jung Kim, Ga-Yeong Lee, Hae-Yeon Cho, Syed Al Jawad Sayem, Eon-Bee Lee, Seung-Jin Lee, Seung-Chun Park

**Affiliations:** 1 Laboratory of Veterinary Pharmacokinetics, Institute for Veterinary Biomedical Science, College of Veterinary Medicine, Kyungpook National University, Daegu 41566, Republic of Korea; syedaleemabbas77@gmail.com (M.A.A.); seafog@hanmail.net (H.-J.K.); ga7464@naver.com (G.-Y.L.); whgodus777@naver.com (H.-Y.C.); aljawadsayem@gmail.com (S.A.J.S.); 2 Department of Aquatic Life Medicine, Pukyong National University, Busan 48513, Republic of Korea; eonbee@pknu.ac.kr; 3 Developmental and Reproductive Toxicology Research Group, Korea Institute of Toxicology, Daejeon 34114, Republic of Korea; 4 Cardiovascular Research Institute, Kyungpook National University School of Medicine, Daegu 41944, Republic of Korea

**Keywords:** fish probiotic, aquaculture, fish gut microbiota, fish nutrition, aquatic microorganisms, *Lactobacillus plantarum*

## Abstract

Aquaculture has grown significantly, contributing to global food security and sustainability; however, intensified fish farming has increased disease susceptibility and antibiotic resistance. This study assessed the probiotic potential of *Lactobacillus plantarum* PSCPL13 (hereafter, PSCPL13), isolated from the intestines of Japanese eels, for enhancing the health of olive flounder. After screening 16 *Lactobacillus* isolates, PSCPL13 was selected because of its potential broad-spectrum antibacterial activity against many pathogens, such as *Vibrio* and *Edwardsiella*. This strain exhibited high acid and bile salt tolerance, which is crucial for intestinal survival. Molecular characterisation confirmed PSCPL13 to be *L. plantarum*. It was resistant to all tested antibiotics and exhibited significant enzyme activity. In vivo assays in olive flounder revealed that the body weight and length of the fish were significantly higher, while the prevalence of pathogens was lower in their gut microbiota. Regarding haematological parameters, the treated groups exhibited lower eosinophil counts and higher IgM levels, suggesting enhancement of the immune system. These findings indicate that PSCPL13 is a potential probiotic that can be used in aquaculture for naturally improving fish health, growth and immunity, in addition to combating antibiotic resistance and reducing environmental impacts. These findings not only highlight the potential of PSCPL13 in sustainable aquaculture but also provide a foundation for the development of future probiotics.

## 1. Introduction

*Lactobacillus* species are commonly used in biotechnology and exhibit various health benefits. Different *Lactobacillus* strains have been developed for use in aquaculture, animal husbandry and other fields. Multiple studies have revealed that their main application in aquaculture is to protect fish from pathogens [1,2,3]. Aquaculture has been fundamental in facilitating sustainable living and ensuring global food security [4]. According to the United Nations Office for the Coordination of Humanitarian Affairs, seafood production for human consumption is increasing. Fisheries and aquaculture production peaked globally in 2020 because of Asia’s aquaculture boom, contributing $151 billion to the global economy. Seafood consumption is estimated to increase by 15% and reach 21.4 kg per person by 2030 because of population growth [5]. New demands for eco-friendly aquaculture necessitate research on aquatic probiotics [6]. Probiotics can enhance production, nourishment and immune health. Moreover, they can help combat disease and overcome drug resistance [7]. They are crucial for growth, enzyme activity, immunity, gut microbial ecology and antibiotic defence [8,9,10], promoting healthy aquaculture [11]. *Streptococcus*, *Edwardsiella* and *Vibrio* species are some pathogens that can adversely impact the growth of farmed fish; they directly contribute to the mortality of farmed fish and shellfish [12,13].

Aquaculture probiotics, such as *Lactobacillus*, *Streptococcus* and *Bacillus* species, help prevent diseases [14]. *Lactobacillus* strains are used to treat *Edwardsiella*, *Aeromonas*, *Vibrio* and *Flavobacterium* infections [12,15]. Lactic acid bacteria (LAB) and *Bacillus* species boost immunity and prevent bacterial infections [16]. Moreover, probiotics support the management of the microbiota and improve host health [17]. Diet, lifestyle and the environment can impact an organism’s microbiota. The gut microbiome of diversely fed fish, such as eels, is highly variable. The aquatic environment determines the fish gut microbiome, as freshwater and migrating eels come into contact with numerous microbes. The fish gut is a microenvironment in itself, with bacteria controlling the microbial ecology [18,19,20].

Probiotics improve animal health and welfare, enhance the stability of aquaculture operations and facilitate healthy, productive operations [21]. In this study, we isolated *L. plantarum* strains and characterised *L. plantarum* PSCPL13 (hereafter, PSCPL13), a novel probiotic strain isolated from normal Japanese eels. We examined the efficacy of PSCPL13 in the gut of olive flounder, including its antibacterial efficacy against fish infection, safety via toxicity tests, effects on fish development and microbiota, adhesion ability and growth in the fish gut. Our findings provide novel strategies for improving immune function and building healthy gut microbiota in aquaculture, potentially enhancing fish health and production efficiency. We also determined the function and activity of PSCPL13 in the intestine of olive flounder by assessing its adherence to the intestinal mucosa and its survival and replication abilities. Our findings suggest that PSCPL13 is an ideal candidate for aquaculture. This strain can not only promote fish growth but also enhance immune response, thereby providing high protection against disease. This dual advantage emphasises its efficacy in increasing aquaculture productivity and health resilience.

## 2. Materials and Methods

### 2.1. Statement on Ethics

All animal experiments were approved by the Institutional Animal Care and Use Committee (IACUC) of Kyungpook National University. The experiments were designed to avoid unnecessary animal labour and minimise any potential for animal pain or suffering during the research.

### 2.2. Isolation and Characterisation of PSCPL13

Healthy Japanese eels (*Anguilla japonica*) not exposed to any probiotics were raised in Hwasun-gun (Jeollanam-do, Republic of Korea) and brought from the fish farm to the laboratory under temperature-controlled conditions. The intestinal contents were aseptically removed and homogenised for processing. The intestines were cultured on De Man–Rogosa–Sharpe (MRS) agar and blood agar (BA) and incubated anaerobically for 24 h at 30 °C. The assessment of Gram stain reaction, morphology, catalase activity, lactic acid activity and haemolytic activity of the isolates revealed distinct findings. The *Lactobacillus isolates* (PSCPL01–16) were Gram-positive, acid-loving, catalase-negative, non-haemolytic bacteria. The selective medium was prepared with 0.5% skim milk, 0.2% methylcellulose, 0.2% corn starch and 0.5% calcium phytase. Isolation was performed using 2% Congo red. Cellulase and α-amylase levels were assessed by washing the samples with 1 M NaCl. Then, isolates testing positive for protease, cellulase, α-amylase and phytase activities were chosen for subsequent analyses.

### 2.3. Molecular Characterisation of PSCPL13

Genomic DNA was extracted using a kit obtained from Qiagen (Hilden, Germany). *Lactobacillus* species were identified by performing 16S rRNA analysis. PCR amplification was performed using 50 pmol of forward (5′-AGA GTT TGA TCC TGG CTC AG-3′) and reverse (5′-AAG GAG GTG ATC CAG CC-3′) primers, 50 ng of DNA, 5 µg of 10× polymerase buffer and 1 U Taq DNA polymerase. The PCR program was as follows: an initial step at 95 °C for 5 min, followed by 30 cycles at 95 °C for 1 min, 55–60 °C for 1 min and 72 °C for 1 min. Nucleotide sequencing was performed using the BigDye^TM^ Terminator Cycle Sequencing Kit (ThermoFisher, Waltham, MA, USA). Sequences were analysed using an ABI PRISM 377 sequencer (Perkin-Elmer, Waltham, MA, USA) after cloning into pSTBlue-1 (Novagen, Madison, WI, USA). The identity of PSCPL13 (KCCM1168P) was confirmed by sequence matching.

### 2.4. Antibacterial Activity of PSCPL13

The minimum inhibitory concentration (MIC) for PSCPL13 was calculated using a microtitre plate assay according to the Clinical and Laboratory Standards Institute (CLSI) protocol, with some modifications [22,23]. The PSCPL13 isolate was compared with *Escherichia coli, E. tarda*, *S. iniae*, *V. anguillarum*, *V. alginolyticus*, *V. parahaemolyticus* and *V. harveyi*, which are known fish pathogens [24,25]. The *Lactobacillus* culture supernatant was added to Luria–Bertani (LB) broth at different ratios: 100%, 50%, 25%, 12.5%, 6%, 3%, 1.5% and 0.7%. Next, 50 µL of bacterial solution [10^5^ colony-forming units (CFU)/mL] was spread onto LB agar plates and incubated at 37 °C for 24 h. After incubation, the samples were serially diluted and bacterial counts (CFU) were obtained.

The antibiotic resistance test was performed to assess the resistance to antibiotics commonly administered to fish and animals through feed or therapy. To perform this test, PSCPL-13 was cultured overnight and then adjusted to a concentration of 10^6^ CFU/mL. Next, 100 μL of this bacterial solution was added to a 96-well plate. A prediluted sample of the test antibiotic Cephalexin, Colistin sulphate, Enrofloxacin, Cefalonium, Amoxicillin trihydrate, Penicillin G procaine, Norfloxacin, Spectinomycin, Tylosin base, Cefuroxine sodium, Florfenicol, Penicillin G benzathine, Gentamicin sulphate, Streptomycin sulphate and DHSM (100 μL) was then added to each well. Subsequently, the plate was incubated at 37 °C and bacterial growth was measured over 24 h to identify the MIC for growth and minimum bactericidal concentration for sterilisation.

### 2.5. Acid and Bile Salt Tolerance

The acid and bile salt tolerance of the *Lactobacillus* isolates and control strains was assessed using a previously described method [26], with slight adaptations based on our study objectives. PSCPL13 and *L. plantarum* KCCM 12116 (hereafter, KCCM 12116) were grown to 0.1 × 10^7^ CFU/mL and added to 5 mL of MRS broth supplemented with 1 N HCl at pH 2.0, 3.0, 4.0, 5.0 and 7.0 (control). Microbes emerged at 0, 0.5, 1 and 8 h post-injection. For assessing tolerance to bile salts, the isolates and control strains (10^5^ CFU/mL) were inoculated at 0.1% into 5 mL of MRS broth containing 0%, 0.1%, 0.3% and 1% bile salts. Tolerance was assessed by performing sampling at 0, 3, 6, 12, 24 and 48 h after inoculation, followed by serial dilution.

### 2.6. Single Oral Toxicity Test in Rats

The experimental setup complied with institutional protocols approved by the institutional animal care and use committee (IACUC) (protocol KNU-2015-0020). Specific pathogen-free (SPF) Sprague–Dawley (SD) rats were procured from Orient Bio (Seongnam, Republic of Korea) and housed in groups of five within cages at a controlled temperature of 22 ± 2 °C and relative humidity of 55 ± 10%. The rats were subjected to a 12-h light–dark cycle, with illumination ranging from 150 to 300 lux. They were provided sterilised feed sourced from Hyochang Science (Seoul, Republic of Korea) and ad libitum access to purified water. Before the commencement of experimental procedures, a 7-day period was allocated for acclimatisation to the laboratory milieu.

The PSCPL13 isolate was grown for 3 days at 30 °C in MRS broth until the stationary growth phase. The cells were then centrifuged at 5000 rpm for 20 min, and the supernatant was scraped out. The resulting pellet was washed with 0.85% NaCl and diluted in MRS broth until a final concentration of 10^11^ CFU/mL was reached. The rats were divided into four groups: PSCPL13-treated groups (10^11^, 10^9^ and 10^7^ CFU/mL) and an untreated control group (administered 0.85% NaCl). For 2 weeks, the rats were administered an oral gavage of 20 mL/kg body weight/day to perform toxicological assessments. Serious surveillance lasted for 6 h on the first day. Daily surveillance was performed to look for signs of toxicity, with euthanasia performed on day 14. On days 1, 3, 7 and 14, temperature and body weight were measured and daily consumption of water and feed was monitored. At the end of the experiment, the rats were anaesthetised with CO_2_; in detail, the rats were euthanised by carbon dioxide inhalation. The lid was placed over the cage. CO_2_ was delivered from the tank at a flow rate of 10–30% per minute. Finally, the animals were monitored for the cessation of respiration and left in the chamber for at least 1 min after respiration had ceased [27,28,29] and blood was pumped in via the abdominal vein. Following euthanasia, all vital organs were observed and sub-weighed.

Toxicity testing is crucial for making public health and regulatory decisions about new treatments. Toxicity testing methods largely rely on laboratory rats, mice and rabbits [30]. Oral toxicity testing in rats helps determine whether a substance is likely to cause harm once consumed orally. This type of experimentation is crucial for identifying the safety profile of substances, thereby facilitating regulatory decisions and protecting health. The use of a rodent model facilitates the extrapolation of results to possible responses and widens the utility of toxicological analyses.

### 2.7. Single Oral Toxicity in Olive Flounder

**Figure 1:**
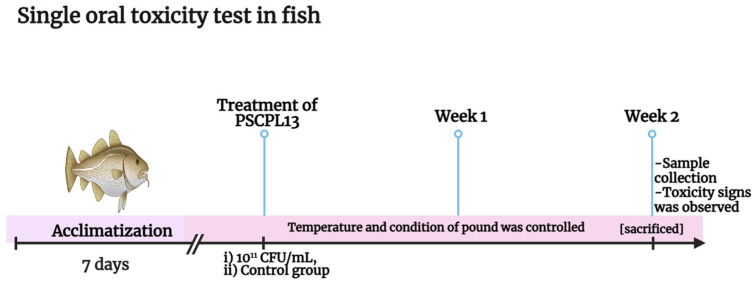
In vivo assessment of the toxicity of *Lactobacillus plantarum* PSCPL13 in fish. The figure provides a flow diagram of the single oral toxicity test in fish.

To determine the toxicity of PSCPL13 in olive flounder, its doses were regulated by mixing with basic feed and wheat flour (Table 1). The experimental feeds consisted of feed protein from sardine fish meal, anchovy fish meal, tankage meal, poultry by-product meal, soybean meal, wheat gluten and soy protein concentrate. They mainly contained flour for carbohydrates and fish oil for lipids. The crude protein and crude lipid contents of all experimental feeds were identical. PSCPL13 was prepared at a concentration of 10^11^ CFU/mL, and any test material left behind was discarded at the end of the experiment. The concentration, stability and homogeneity of the test substance were not assessed independently. The experiment was conducted on 20 *Paralichthys olivaceus* adults with an average weight of 300 g at a halibut farm (Yongcheon Fisheries, Yongcheon, Republic of Korea) on Jeju Island. The tested fish, as well as the fish that were bred for the test, were kept in a little tank at the halibut farm a week before the experiment and then trained for another week. A prototype from Clean Bio Co., Ltd. Daejeon, Republic of Korea was utilised as the test specimen. In the test group, the fish were orally administered the PSCPL13 solution (10^11^ CFU/mL). The average mortality rate over 14 days was then calculated (Figure 1). For the entire test period, the number of deaths was recorded daily after immunisation. The presence of abnormal symptoms (e.g., equilibrium disorders, irregular swimming, bleeding, respiratory disturbances, colour changes and spinal paralysis) was also recorded.

The water temperature during the challenge ranged from 20 °C to 25 °C, while the water temperature for 2 weeks after the challenge was 25 ± 1 °C. All water parameters remained unchanged throughout the experiment: the ammonium (NH_3_/NH_4_) concentration ranged from 0 to 0.25 mg/L, nitrogen dioxide (NO_2_) concentration ranged from 0.3 to 0.8 mg/L, pH ranged from 8.0 to 8.2 and DO concentration ranged from 6.7 to 7.8 mg/L. The water in the experiment was kept at 33–35 ppt, which is the natural seawater temperature. Salinity was measured once a week with a calibrated refractometer to make sure that it was constant and correct throughout the adaptation and challenge phases. The experiment was performed to verify the toxicity of the target probiotic. This limit test was performed using 10^11^ CFU/mL. The cumulative mortality rate of 20 adult olive flounders (average weight, 300 g) was assessed over 14 days.

### 2.8. Effects of PSCPL13 Probiotics on Aquaculture Productivity

PSCPL13 was cultured on the MRS medium at 30 °C for 48 h, followed by the addition of feed additives (Clean Bio, Daejeon, Republic of Korea) and drying at 30 °C. Olive flounders (*P. olivaceus*) used in this research were bought from Dongil Fisheries (Gangneung, Republic of Korea); their average weight was 69.2 g (+). The fish were fed standard fish feeds before the experiment.

In a large breeding program, the fish were randomly assigned to a probiotic-treated group (*n* = 1000) and an untreated control group (*n* = 1000). Each group received feed twice daily for 3 months and was swabbed biweekly (*n* = 10; three replicates) for the duration of the study. The prototype-treated group received 100 kg of feed containing 500 g (10^11^ CFU/g) of PSCPL13 additives (total daily feed requirement). On the other hand, the control group received only 100 kg of feed. The optimal dose was determined based on our prior research, which included oral pharmacokinetic analyses [31], as well as the oral toxicity test in olive flounder conducted in the present study.

The weight and size of the fish were recorded every 2 weeks. On the last day of the experiment, the average weight and length were calculated by sampling the fish from both groups. In brief, 10 fish were randomly selected from each group, thrice in total. Their average weight and length were then calculated. The values between the treated and control groups were compared. At the end of the experiment, five randomly selected fish from each group were euthanised, and their gut microbiota was analysed through microbiota profiling.

#### 2.8.1. Medium Plating and Culture

Intestinal bacteria were counted by culturing them on BA (5% sheep blood on tryptic BA base; Difco, Detroit, MI, USA) under both anaerobic and aerobic conditions. MacConkey agar (Difco) and *Lactobacillus* MRS broth (Difco) were used to culture Enterobacteriaceae species and LAB, respectively. The solid medium was divided into four sections, and 50 µL samples (10^1^, 10^3^, 10^5^ and 10^7^) CFU were applied to each section. The samples were incubated at 30 °C both aerobically and anaerobically (in an aerobic jar), and the number of colonies was counted after 48 h. Bacterial counts in bowel and intestinal tract contents were determined by diluting with 0.09% NaCl. The bacteria were cultured on tryptic BA containing 5% sheep blood and incubated aerobically and anaerobically. On MacConkey and MRS agars, the cells were cultured at 30 °C for 48 h. Analyses were performed in accordance with the ISO 15214:1998 method [32].

#### 2.8.2. Comparative Analysis of Intestinal Bacterial Community

During the testing period, five rats were randomly selected from each group. Their intestines were harvested with care, and the number of bacteria was counted. Specifically, only the bowel contents were extracted and placed into a 50 mL tube. These digested intestinal contents were then subjected to a range of dilutions (10^1^–10^7^) with physiological saline (0.9% NaCl, ordinary saline). The diluted contents were then sterilised and stored in E-tubes. DNA samples from the isolated gut contents were analysed using the QIAamp DNA Stool Kit (Qiagen) to determine changes in the intestinal microbiota. The DNA samples were transferred to Chun Lab, Seoul, Republic of Korea, and intestinal bacterial samples were subjected to metagenomic analyses by performing next-generation sequencing (NGS).

#### 2.8.3. Blood and Immunological Tests

Blood was collected into EDTA tubes using syringes. The blood samples were centrifuged at 8000 rpm for 10 min, following which plasma was isolated and stored at −80 °C. The total white blood cell (WBC), neutrophil (NEU), lymphocyte (LYM), monocyte (MONO), eosinophil (EOS), basophil (BASO) and red blood cell (RBC) counts; haemoglobin (HGB) level; haematocrit (HCT); mean corpuscular volume (MCV); mean corpuscular haemoglobin (MCH) and mean corpuscular haemoglobin concentration (MCHC) were determined. Fish IgM levels were measured using the Biotech ELISA Kit (Cusabio, Wuhan, China). Plasma was used for the analysis in accordance with the guidelines provided in the kit. Immunological tests were performed using blood samples collected in tubes containing heparin. After separating plasma using a vacuum pump, 500 µL of blood was collected in an E-tube and centrifuged at 8000 rpm for 10 min. The separated plasma was used for calculating fish IgM levels.

### 2.9. Statistical Analysis

All statistical computations were performed using SAS 9.4. Pairwise and multiple comparisons between experimental groups were performed using Student’s *t*-test and one-way ANOVA, respectively. Significant differences between the treated and untreated control groups were assessed at a *p*-value of <0.05.

## 3. Results

### 3.1. Isolation and Identification of Probiotics

**Figure 2:**
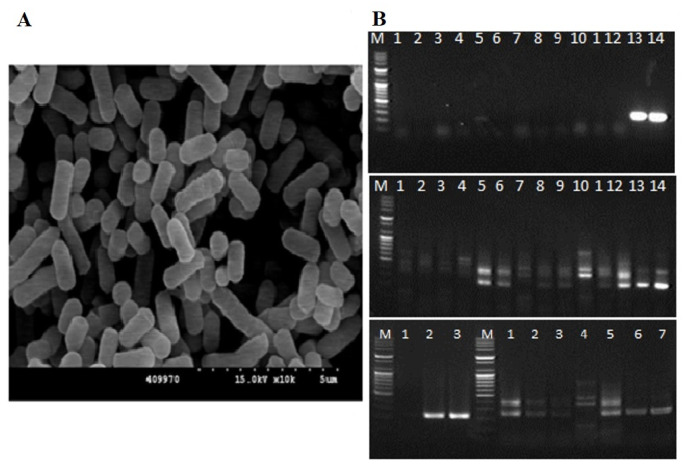
Isolation and characterisation of *Lactobacillus plantarum* PSCPL13. (**A**) Scanning electron microscopy of *Lactobacillus plantarum* PSCPL13. (**B**) PCR bands of isolated *Lactobacillus plantarum* PSCPL13.

In total, 16 *Lactobacillus* strains, namely PSCPL01–16, were isolated from the intestines of Japanese eels. A selective medium was used to distinguish between these isolates. Secondary biochemical tests (assessment of protease, cellulase, α-amylase and phytase activities) were also performed. The isolates selected through these analyses were PSCPL11, PSCPL13 and PSCPL16. PSCPL13 ranked highest in the downstream analysis conducted using scanning electron microscopy (Figure 2). Table 2 presents the overall characteristics and biochemical features of all 16 strains. Classification was performed based on the Gram stain reaction, cell morphology, catalase activity, acid production, lactic acid production, ability to haemorrhage on BA and enzyme activities. Notably, PSCPL13 was a Gram-positive, rod-shaped, catalase-positive bacterium that produced both D and L forms of lactic acid. It did not haemolyse on BA. Moreover, it produced large amounts of protease, cellulase, α-amylase and phytase.

### 3.2. Molecular Characterisation

**Figure 3:**
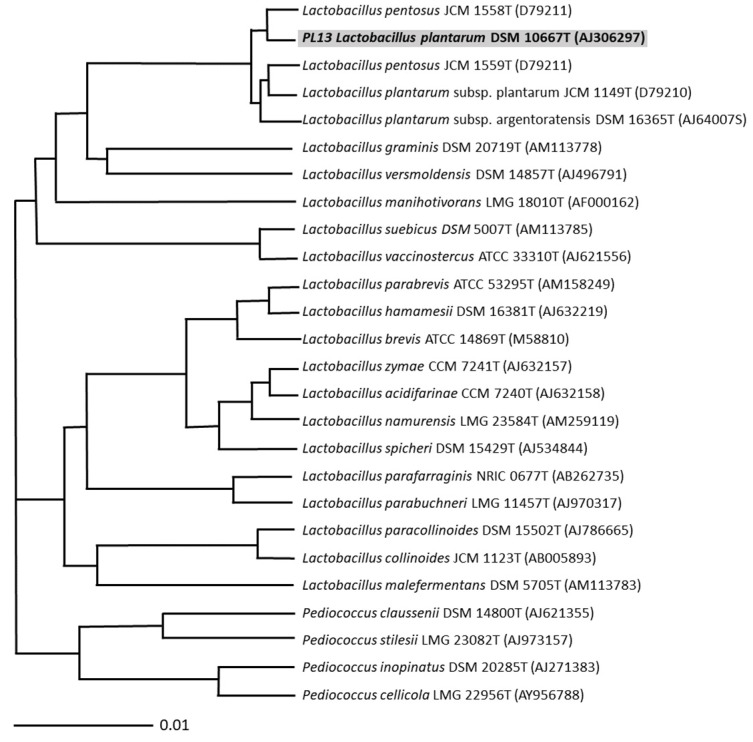
Characterisation of *Lactobacillus* isolates by 16S rRNA gene sequencing.

Our 16S rRNA gene sequencing revealed PSCPL13 to be *L. plantarum* based on a 99% identity match (Figure 3). The discovered strain was named PSCPL13 and enlisted in the Korean Culture Centre of Microorganisms under the identification code KCCM11682P and the Korean patent number KR1020150093685A. Our 16S rRNA gene sequencing thus enabled the identification and characterisation of a single *Lactobacillus* strain, which was then maintained in a microbial culture laboratory for further research.

### 3.3. Antibacterial Activity, Bile Salt Tolerance and Acid Tolerance of PSCPL13

**Figure 4:**
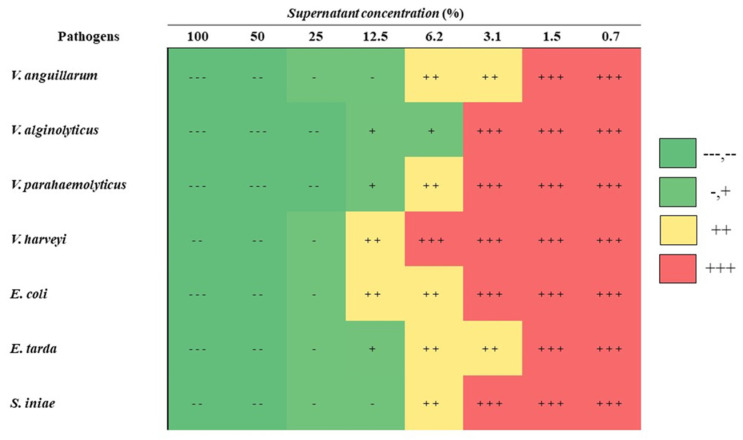
Antibacterial activity of PSCPL13 against fish pathogens. Symbol descriptions: No growth (−−−), <10^2^ CFU/mL (−−), <10^3^ CFU/mL (−), <10^5^ CFU/mL (+), <10^7^ CFU/mL (++) and <10^8^ CFU/mL (+++).

The antimicrobial activity of PSCPL13 was assessed using seven fish pathogens: *V. anguillarum*, *V. alginolyticus*, *V. parahaemolyticus*, *V. harveyi*, *E. coli*, *E. tarda* and *S. iniae*. As shown in Figure 4, PSCPL13 exhibited antibacterial activity against all these pathogens. Specifically, a 50% probiotic supernatant of PSCPL13 inhibited the growth of *V. alginolyticus* and *V. parahaemolyticus*, while a 100% probiotic supernatant completely inhibited the growth of *E. coli*, *E. tarda* and *V. anguillarum*. Notably, a significant difference existed between the treated and control groups. In summary, PSCPL13 exhibits strong antibacterial activity against fish pathogens and may be suitable for use as a probiotic in the aquaculture sector.

Table 3 summarises the results of the antibiotic resistance test. Remarkably, PSCPL13 survived in the presence of most of the tested drugs. Its resistance was as good as or better than that of traditional LAB. In particular, PSCPL13 was highly resistant to cephalexin, colistin sulphate, norfloxacin, gentamicin sulphate and streptomycin sulphate, with MIC and MBC values being greater than 256 µg/mL. In contrast, it was slightly less resistant to enrofloxacin, amoxicillin trihydrate, tylosin base, cefuroxime sodium, florfenicol, penicillin G procaine, penicillin G benzathine and dihydrostreptomycin (DHSM), with MIC and MBC values being between 2 and 64 µg/mL. Together, these findings indicate that PSCPL13 is a potential probiotic strain that can maintain its efficacy in feeds containing certain antibiotics.

**Figure 5:**
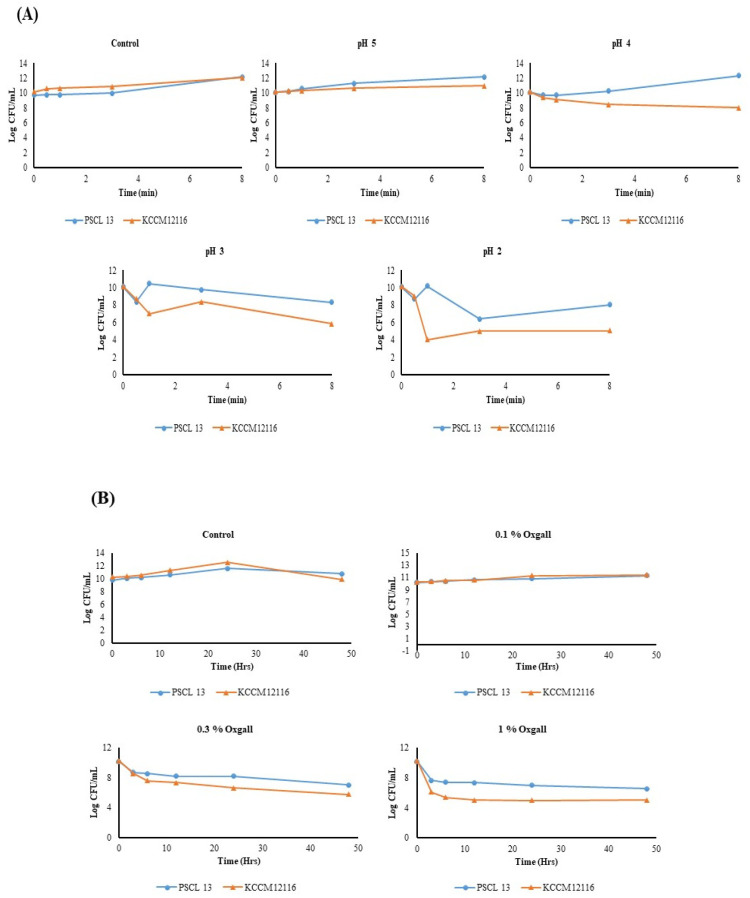
(**A**) Acid resistance and (**B**) bile salt tolerance of *Lactobacillus plantarum* PSCPL13.

PSCPL13 was much more acid-tolerant than KCCM 12116. In particular, the number of PSCPL13 cells dramatically increased after 8 h of incubation at pH 2.0 with KCCM 12116. At pH 2.0, the concentration of PSCPL13 cells increased from 2.5 × 10^8^ CFU/mL at 0 h to 2.2 × 10^6^ CFU/mL at 8 h. In contrast, the concentration of KCCM 12116 cells decreased from 3.0 × 10^8^ CFU/mL at 0 h to 2.4 × 10^3^ CFU/mL at 8 h. This finding suggests that PSCPL13 can flourish and remain alive in more acidic conditions than KCCM 12116. Moreover, the concentration of PSCPL13 cells was higher than that of KCCM 12116 cells at pH 3.0 and 4.0 (albeit not as high as that at pH 2.0). Interestingly, both strains grew similarly at pH 5.0. PSCPL13 was more acid resilient than KCCM 12116, particularly at pH 2.0, 3.0 and 4.0 (Figure 5A). These findings can facilitate the selection of microbial strains for probiotic formulations because acid tolerance is a key determinant of probiotic survival and effectiveness in the gut.

PSCL13 did not react to 1% bile salts for 48 h; it exhibited a high tolerance to bile salts. The number of bile salts also influenced the survival rate, with a large logarithmic difference (log 2) being noted between the treated and control groups after 24 h in the presence of higher bile salt levels. On the other hand, KCCM 12116 was less tolerant to bile salts; it failed to survive upon exposure to 1% bile salts for more than 24 h. The amount of bile salts also determined the survival of this strain. Its survival was significantly reduced in the presence of 0.3% bile salts. PSCL13 exhibited higher tolerance to bile salts; it survived for up to 48 h in the presence of 1% bile salts. In contrast, KCCM 12116 was non-viable after being exposed to 1% bile salts for more than 24 h. In the case of PSCL13, after 24 h of exposure to higher levels of bile salts, a larger logarithmic difference in survival was noted between the treated and control groups (Figure 5B). These data suggest that PSCL13 is more suitable than KCCM 12116 in systems with higher levels of bile salts.

### 3.4. Single Oral Toxicity Test of PSCPL13 in SD Rats

**Figure 6:**
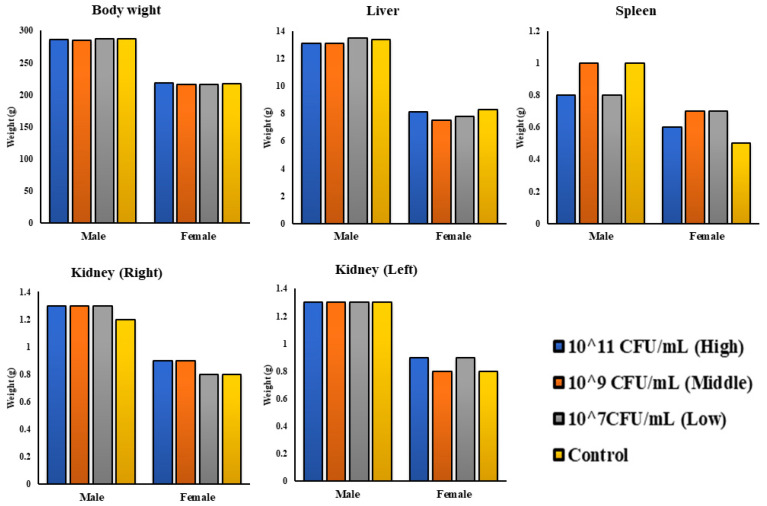
Absolute organ weights (g) of male and female rats following oral administration of *Lactobacillus plantarum* PSCPL13.

The toxicity of PSCPL13 in rats was also analysed in this study. For 2 weeks, the rats were orally administered three different concentrations of PSCPL13. The control group received only 0.85% NaCl. The health of the rats was constantly monitored during the study period. No rat died in any treated group. Moreover, no evidence of treatment-related illness or toxicology appeared over the 14-day experimental period. No significant differences were noted in body weight, temperature, water intake or feed intake between the treated and control groups. Furthermore, upon being euthanised, neither body temperature nor organ mass significantly differed between male and female rats (Figure 6). These results indicate that PSCPL13 exhibits no adverse effect or toxicity in rats, which is a key measure of probiotic safety. It is therefore safe to be administered orally to rats.

### 3.5. Single Oral Toxicity Test in Olive Flounder

**Figure 7:**
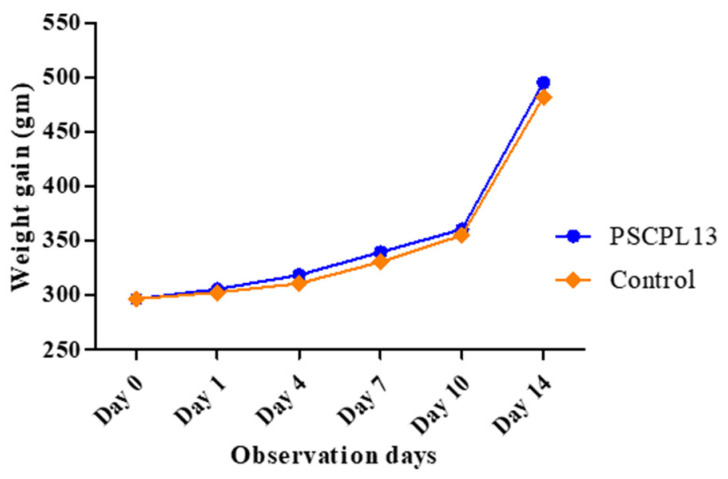
Body weight changes in olive flounder after single-dose administration.

No death or clinical symptom was noted in fish exposed to ≥10^11^ CFU/mL PSCPL13. However, body mass was significantly higher in the treated group than in the control group. The treated group gained 198.8 g of body mass, while the control group gained 185.6 g. The percentage of weight gain was 7% in the treated group (Figure 7). Water quality conditions were monitored over the study period. Small changes were noted in pH levels, DO concentrations and nitrogen compound concentrations. However, all measured values fell within the normal and acceptable limits (Table 4).

### 3.6. Effects of PSCPL13 Probiotics on Fish Farming

**Figure 8:**
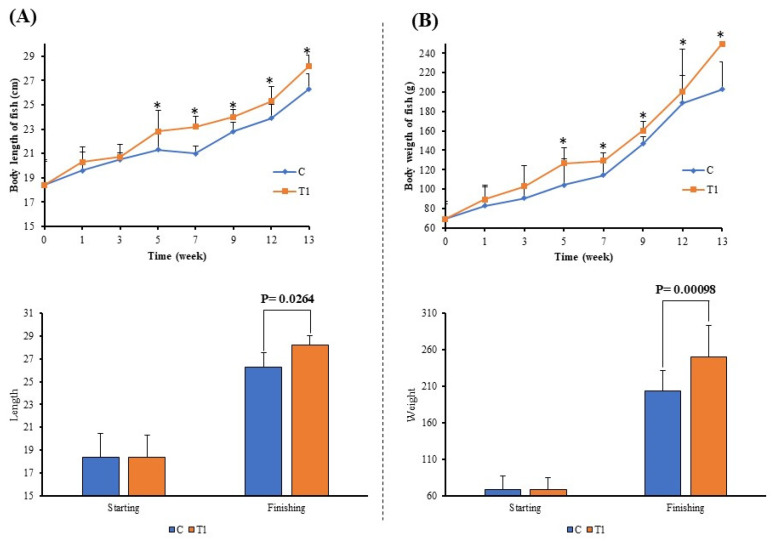
(**A**) Body length and (**B**) weight changes in olive flounder after treatment with *Lactobacillus plantarum* PSCPL13. The asterisk (*) in the figure represents the statistically significant difference between the control and treatment groups at the appropriate time points. Significance was tested by *t*-test, * is for *p* < 0.05.

Assessment of the growth of olive flounder revealed that the fish treated with PSCPL13 were much longer and heavier than the control fish. After 13 weeks, the average body length was 28.2 ± 0.9 cm in the treated group and 26.1 ± 1.3 cm in the control group. The fish in the treated group significantly increased in size only after 5 weeks of treatment, indicating that PSCPL13 had a growth-enhancing effect. As shown in Figure 8A, the body length considerably differed between the treated and control groups. The FCR of the fish in the treated group was approximately 1.392, which is considered to be good [33,34].

As shown in Figure 8B, the treated group exhibited significantly higher weight gain than the control group. These results indicate that PSCPL13 can be used to improve not only the growth but also the body mass of olive flounder. The growth rate in the treated group was approximately 8.1%, while that in the control group was 5.7%. The difference in weight gain was obvious, as the treated group gained approximately 18.7% more weight than the control group. These findings indicate that PSCPL13 can be used as a growth stimulant for olive flounder. Major improvements were noted in body length and weight. Thus, this treatment may have important applications in aquaculture for increasing the productivity and profitability of olive flounder production.

#### 3.6.1. Medium Plating and Culture

**Figure 9:**
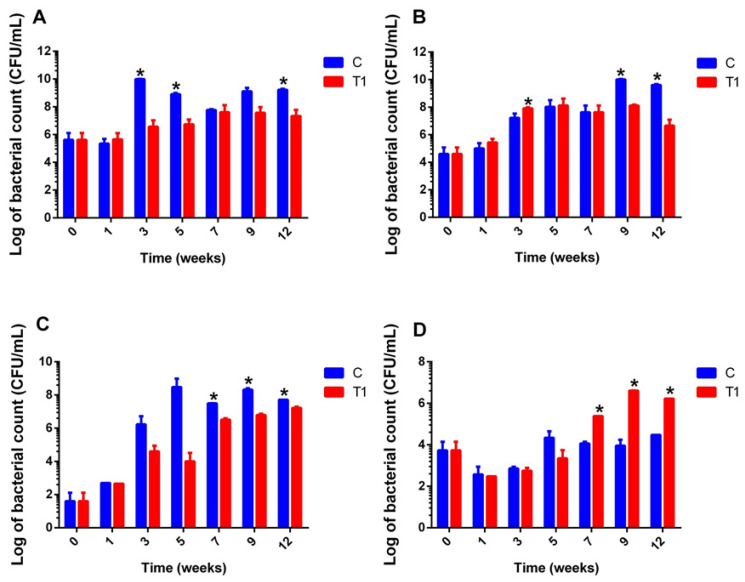
Counts (colony-forming units) of different bacteria on different agar plates under anaerobic and aerobic conditions. (**A**) Bacterial growth under anaerobic conditions. (**B**) Bacterial growth on blood agar under aerobic conditions. (**C**) Bacterial growth on MacConkey agar under aerobic conditions. (**D**) Bacterial growth on MRS agar under aerobic conditions. The asterisk (*) in the figure represents the statistically significant difference in bacterial count between the control and treatment groups at the appropriate time points. Significance was tested by *t*-test, * is for *p* < 0.05.

The administration of PSCPL13 significantly reduced the population size of pathogenic bacteria on both BA and MacConkey agar. Large differences in bacterial counts were noted 7 weeks after culture on MacConkey agar and 9 weeks after culture on BA under aerobic conditions (Figure 9B,C). Under anaerobic conditions, bacterial counts decreased by week 7, with the decrease being statistically significant by week 12 (Figure 9A). Compared with the control group, a steady and statistically significant increase in the total *Lactobacillus* count was noted from 7 to 12 weeks post-treatment in the treated group (Figure 9D).

#### 3.6.2. Comparative Analysis of Intestinal Bacterial Community

**Figure 10:**
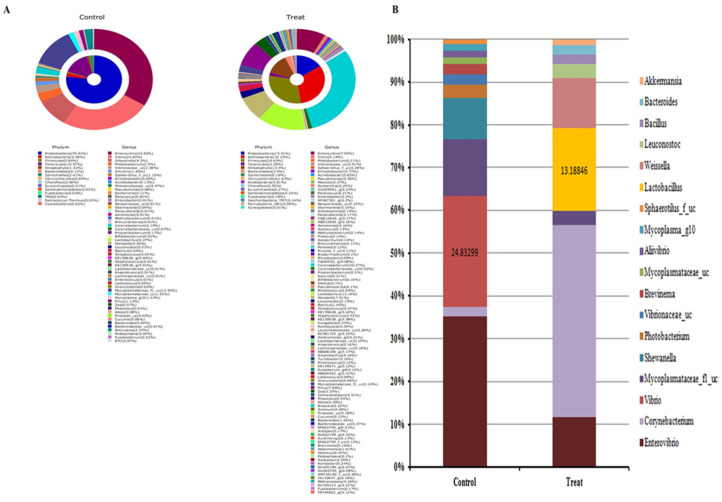
Effects of PSCPL13 on the gut microbiota of olive flounder. (**A**) Comparison of variations in the gut microbiota of olive flounder. (**B**) Relative percentages of microbes. The bar indicates the relative abundance of each bacterial genus. The fish in the control group were not treated with PSCPL13, whereas those in the treated group were administered PSCPL13.

Metagenomic analyses revealed that the prototype-fed test population consisted of 16 phyla and 96 genera. The control population, on the other hand, consisted of 15 phyla and 52 genera (Figure 10A). To obtain more information on how the intestinal bacteria changed in the prototype-fed test population, the identified bacterial colonies were normalised to 100% at the genus level and compared; genera accounting for less than 1% of the total bacterial content were excluded. The genus *Lactobacillus* accounted for 13.2% of the total bacterial content in the treated group. In comparison, LAB were absent or accounted for less than 1% of the total bacterial content in the control group. Furthermore, the control group was heavily populated with *Vibrio* (a bacterial pathogen), which accounted for as much as 24.83% of the total bacterial content. However, the genus *Vibrio* was much less prevalent and accounted for less than 1% of the total bacterial content in the treated group (Figure 10B).

#### 3.6.3. Blood and Immunological Tests

**Figure 11:**
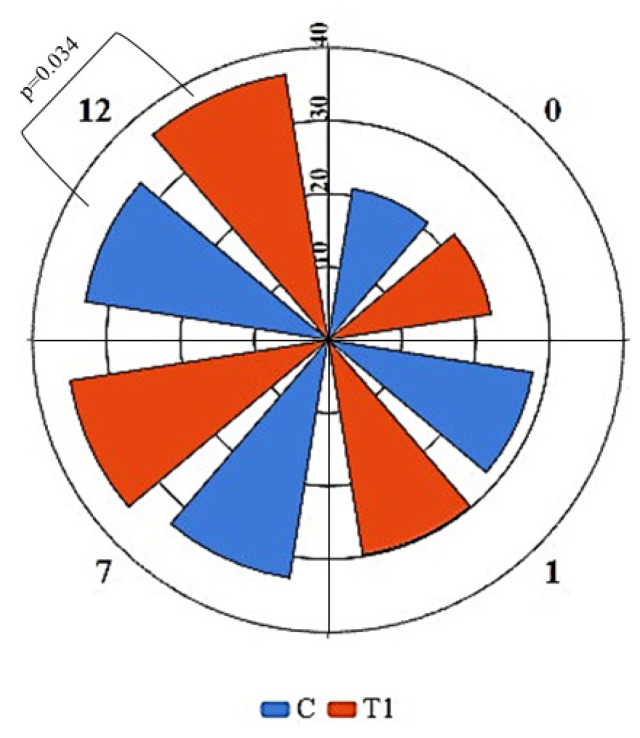
Effect of PSCPL13 on IgM levels in olive flounder.

A full blood profile was performed, including WBC, NEU, LYM, MONO, EOS, BASO and RBC counts; HGB level; HCT; MCV; MCH; MCHC; red cell distribution width (RDW); platelet (PLT) count; mean platelet volume (MPV); platelet crit (PCT) and platelet distribution width (PDW). The values were similar in both treated and control groups. However, the EOS count, which tends to spike in the presence of external infections, such as parasitic infestations, was 27.2% on average in the control group; this was almost twice the value in the treated group (14%) (Table 5). The EOS count was thus lower in the treated group than in the control group. The IgM level was somewhat higher in the treated group (41.64 g/mL) than in the control group (40.16 g/mL) (Figure 11); however, no significant differences were noted between the treated and control groups. These results are presented in Table 5 and Figure 11.

## 4. Discussion

Given the demand for fish, fish farming has increased dramatically. However, this intensification has led to more fish diseases that can cause devastating commercial losses [35,36]. Bacterial infections pose a problem in aquaculture and must be effectively managed to meet the demand. Antibiotics are commonly used to treat bacterial infections; however, the presence of drug-resistant bacteria and environmental pollutants poses a risk to human health. The use of probiotics for regulating the gut microbiome is a viable solution to reduce diseases in aquaculture [37,38].

Probiotics play a crucial role in aquaculture. The right strains with broad tolerance and synergistic efficacy can kill pathogens, build immunity, control gut microbes, minimise antibiotic use and resistance and enhance sustainability [39,40,41]. Our experiments were designed to reduce the need for antibacterial treatments in aquaculture and increase fish growth and disease resistance. For this purpose, a new probiotic was isolated and characterised from Japanese eels. The effectiveness of this probiotic against pathogens of olive flounder was then assessed. Initially, 16 *Lactobacillus* strains were isolated from the intestines of Japanese eels. Of these, PSCPL13 was chosen because it is a Gram-positive, rod-shaped bacterium with protease, cellulase, α-amylase and phytase activities. Moreover, 16S rRNA gene sequencing revealed that PSCPL13 was 99% identical to *L. plantarum*. LAB are used as probiotics in aquaculture to protect against the infiltration of pathogens [39]. Most LAB are probiotic, and the application of these novel agents would be extremely beneficial to the aquaculture sector.

Numerous studies have reported the antibacterial activity of probiotics [39,42,43,44,45,46]. In this study, PSCPL13 exhibited strong antibacterial activity against key fish pathogens. Notably, its culture supernatant destroyed all tested pathogens with a success rate of up to 25%. PSCPL13 reduced the growth of *Vibrio* species, *E. coli*, *E. tarda* and *S. iniae*. Our findings support previous findings that emphasised the importance of *Bacillus* species for destroying fish pathogens [16].

In addition to exhibiting antibacterial effects in vitro, PSCPL13 reduced the number of pathogenic bacteria in olive flounder on BA and MacConkey agar plates under both aerobic and anaerobic conditions. This observation also confirms the antibacterial activity of PSCPL13 against pathogenic bacteria, including *Vibrio* species. Moreover, PSCPL13 supported the growth of beneficial bacteria, such as LAB. Supplementation with probiotics increased the percentage of *Lactobacillus*, *Marinilactibacillus* and *Globicatella* species in olive flounder [47]. This pattern was also confirmed through microbiota analysis.

Various tests, such as acid and bile salt tolerance tests, are often performed to determine the susceptibility of *Lactobacillus* probiotics [48]. In our study, *Lactobacillus* species could exist and thrive in the highly restrictive microenvironment of the gastrointestinal tract. Our assessment of the acid tolerance of *L. plantarum* isolates revealed very poor growth at low pH levels.

Furthermore, PSCPL13 could withstand acidic pH levels as low as 2.0. The growth of PSCPL13 was reduced by 70% at pH 2.0, while that of KCCM 12116 was reduced by 61%. PSCPL13 was thus more sensitive to acidic conditions than KCCM 12116. Similarly, previous studies have revealed that *Bacillus* species can tolerate pH levels below 2.0 [49,50]. At pH 3.0, both PSCPL13 and KCCM 12116 grew well. The isolates also grew more vigorously than the reference strain at pH 4.0 and 5.0, suggesting a greater ability to withstand acidity. Acid tolerance is a key probiotic trait that allows a strain to travel across the gut, grow in the intestine and exhibit its antibacterial effects [51].

PSCPL13 also tolerated high levels of bile salts. The bile salt tolerance test revealed that both *L. plantarum* isolates could tolerate up to 0.3% bile salts. However, the growth of both isolates was greatly stimulated in the presence of 1% bile salts. In the presence of 1% bile salts, PSCPL13 survived for 2 days and outperformed KCCM 12116. This outcome was better than that reported in a previous study, in which *L. pentosus* B281, *L. paracasei* substrain *paracasei* E94 and *L. rhamnosus* exhibited tolerance to the 0.5% bile salt solution [50]. The ability of *Lactobacillus* species to withstand bile salts is crucial because bile salts are present in the gut system of organisms and can damage and inhibit the growth of lactobacilli. Taken together, our findings indicate that PSCPL13 exhibits high probiotic activity because of its tolerance to acid as well as bile salt stress.

The toxicity of PSCPL13 in rats was assessed by orally administering the probiotic at three concentrations over 2 weeks. No death, morbidity or toxicity was noted in any treated group. Moreover, body weight, temperature, water intake or feed intake notably varied between the treated and control groups. These findings indicate that the administration of PSCPL13 is non-toxic to rats. A lack of clinical data regarding discolouration, organ size alteration or death supports the in vivo safety of PSCPL13. This probiotic did not affect the intake of feed or water. The treated groups grew proportionally heavier and larger, indicating that the probiotic treatment had a lasting effect. Moreover, the assessment of euthanised rats revealed that PSCPL13 did not exhibit any adverse effects on their body temperature and organ weight. A higher dose of PSCPL13 was thus not harmful to the rats. These findings indicate that PSCPL13 has no side effects or toxicity in rodents, which is crucial for the development of safe probiotics. In summary, these in vivo findings illustrate that PSCPL13 is non-toxic and non-lethal when administered orally to rodents. This is just one of the emerging reports indicating that probiotics can be very beneficial to health when used correctly, without exhibiting any side effects or toxicity.

Similar to the present findings, our previous in vitro findings [52] indicated that PSCPL13 is a promising probiotic candidate. The in vitro pH as well as bile salt tolerance of the strain was validated in the present study. A remarkable fraction of intravenous cells could navigate gastric acid and reach the intestines in a functional form. They could survive in the intestines for hours. Fluoroscopy revealed the fixation of PSCPL13 to the intestinal mucosa at all doses studied. This fixation is essential for a probiotic strain to survive, replicate and function. A lower dose had a higher recovery rate and a sufficiently long half-life, while a higher dose had an extended half-life and was present for the entire study period. 

Our assessment of the growth and survival of olive flounder in the presence of PSCPL13 revealed significant increases in body length and weight in both treated and control groups. The treated group grew faster at only 7 weeks, suggesting that PSCPL13 is a growth enhancer that can improve olive flounder yield and aquaculture profitability.

Moreover, PSCPL13 substantially depleted pathogenic bacterial colonies on blood and MacConkey agar plates, suggesting that it can act as a probiotic and encourage the development of beneficial bacterial communities in the intestinal system. Metagenomic analyses revealed that the fish in the treated group contained more *Lactobacillus* and less *Vibrio* (a usual bacterial pathogen) in their body than those in the control group. PSCPL13 can thus regulate the intestinal bacterial population in olive flounder by encouraging the growth of beneficial bacteria and preventing the growth of pathogenic ones.

In this study, the growth-enhancing, probiotic and immunomodulatory effects of PSCPL13 were assessed. The treated group had a lower EOS count than the control group. The EOS count usually increases during external infections (such as parasitic infections) [53,54]. Thus, PSCPL13 may help olive flounder have stronger immune responses and be less vulnerable to pathogens. In addition, the treated group contained higher levels of IgM, an immunoglobulin that belongs to the humoral immune system, than the control group [55,56]. These findings indicate that PSCPL13 plays an important role as a growth promoter, probiotic and immunomodulator in olive flounder.

**Figure 12:**
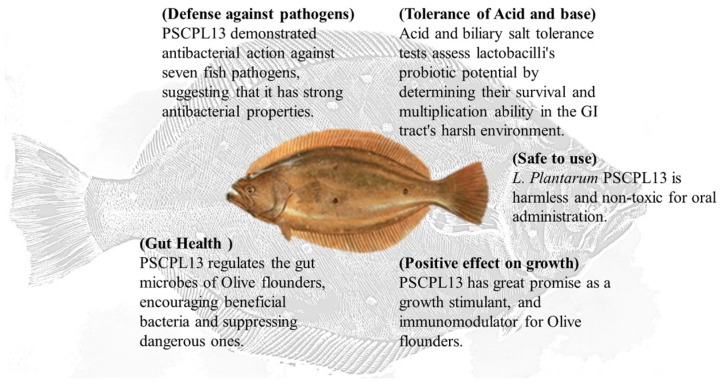
Overview of the beneficial effects of *Lactobacillus plantarum* PSCPL13.

PSCPL13 is a new probiotic strain with high antibacterial activity against fish pathogens and high tolerance to acid and bile salts. These features make it an attractive candidate for reducing fish diseases, avoiding the use of pharmaceuticals and increasing growth efficiency in aquaculture (Figure 12). However, further research is warranted to understand the mechanism underlying the antibacterial effects of PSCPL13 and to optimise treatment protocols. Nevertheless, our results have major implications for aquaculture because the use of PSCPL13 may increase the yield, profitability and sustainability of olive flounder, in addition to reducing the need for antibiotics and other synthetic growth promoters.

## 5. Conclusions

Globally, the increasing demand for fish has also increased fish diseases, resulting in the widespread use of antibiotics in the aquaculture sector. In this study, PSCPL13 was found to exhibit antibacterial effects. It could kill some important fish pathogens, such as *Vibrio* species and *E. coli*. The higher inhibitory activity of this strain against pathogens indicates its potential for preventing diseases in aquaculture systems. Furthermore, oral toxicity tests in SD rats and olive flounder established the safety and biocompatibility of PSCPL13, with no evidence of toxicity even at very high doses. This is a crucial property for its use in animal feeds and probiotic formulations. This probiotic exhibited high antibacterial activity against fish infections and was safe to use in fish. In aquaculture trials, PSCPL13 administration significantly improved the growth performance of olive flounder. It increased the body weight and length of the fish, as well as enhanced feed conversion efficiency. The ability of PSCPL13 to reduce pathogenic bacterial populations and promote beneficial microbial genera, such as *Lactobacillus*, underscores its dual role in enhancing gut health and reducing disease risk. Metagenomic analyses further confirmed these findings by demonstrating substantial shifts in the gut microbiome toward a more favourable composition, characterised by reduced pathogen prevalence and increased probiotic abundance. The use of this probiotic in fish farms can minimise antibiotic use. Thus, PSCPL13 has great potential to play a valuable part in improving global aquaculture sustainability, while preserving fish health, human health and the environment.

## Figures and Tables

**Table 1:** Composition of the basal diet of the olive flounder *Paralichthys olivaceus* (%, dry matter).

Component	Control	Test
Sardine fish meal (FM)	31.5	31.5
Anchovy FM	31.5	31.5
Soybean meal	12	12
Wheat flour	16.9	0
Wheat gluten	1	1
Fish oil	3.4	3.4
Lecithin	0.5	0.5
Betaine	-	-
Monocalcium phosphate	0.5	0.5
Mineral mix	1	1
Vitamin mix	0.8	0.8
Vitamin C	0.3	0.3
Vitamin E	0.3	0.3
Choline	0.3	0.3
Wheat flour containing PSCPL13	0	16.9
Total	100	100
Crude protein (%)	53.5	52.8
Crude Lipid (%)	8.5	8.6
Gross Energy (kcal/g)	4.75	4.74
Metabolisable Energy (kcal/g)	4.2	4.1
Nitrogen-Free Extract (%)	60	61
Dry Matter (%)	89	89

**Table 2:** Overall characteristics and biochemical properties of intestinal bacteria isolated from *Anguilla japonica* eels.

Isolate	Gram Stain Reaction	Cell Morphology	Catalase Activity	Acid Production	Lactic Acid Form	Haemolysis	Skim Milk Test (Protease Activity)	Methylcellulose Test (Cellulase Activity)	Corn Starch Test (α-Amylase Activity)	Ca-Phytate Test (Phytase Activity)
PSCPL01	+	Round	+	+	(−)	+	(−)	+	+	(−)
PSCPL02	+	Rod	+	+	D, L	+	(−)	(−)	+	(−)
PSCPL03	+	Rod	+	(−)	L	+	++	(−)	+	+
PSCPL04	+	Round	(−)	(−)	L	+	(−)	(−)	(−)	(−)
PSCPL05	+	Rod	(−)	(−)	D	+	+	(−)	(−)	+
PSCPL06	+	Round	(−)	+	D	(−)	(−)	(−)	(−)	+
PSCPL07	(−)	Round	+	+	D	+	++	(−)	+	+
PSCPL08	+	Rod	(−)	(−)	D	(−)	++	(−)	(−)	(−)
PSCPL09	+	Rod	(−)	(−)	D	+	+	(−)	+	(−)
PSCPL10	+	Rod	(−)	+	D, L	+	(−)	(−)	+	(−)
* PSCPL11	+	Rod	(−)	+	D, L	(−)	+++	+	+	+
PSCPL12	+	Rod	(−)	+	D	(−)	+	(−)	+	(−)
PSCPL13	+	Rod	(−)	+	D, L	(−)	+++	++	++	+
PSCPL14	+	Rod	(−)	+	D, L	(−)	+	+	+	(−)
PSCPL15	(−)	Round	(−)	+	L	(−)	+	+	(−)	+
PSCPL16	+	Rod	(−)	+	D, L	(−)	+	+	+	+

(+) = Positive; (−) = Negative; * PSCPL11 = *Lactobacillus pentosus* PL11.

**Table 3:** Minimal inhibitory concentrations and minimal bactericidal concentrations of antibiotics against PSCPL13.

Antibacterial Agent	PSCPL13	
	MIC (μg/mL)	MBC (μg/mL)
Cephalexin	>256	>256
Colistin sulphate	>256	>256
Enrofloxacin	8	8
Cefalonium	64	32
Amoxicillin trihydrate	4	2
Penicillin G procaine	32	32
Norfloxacin	>256	>256
Spectinomycin	128	128
Tylosin base	2	8
Cefuroxine sodium	4	32
Florfenicol	4	8
Penicillin G benzathine	4	8
Gentamicin sulphate	128	128
Streptomycin sulphate	64	128
DHSM	64	256

**Table 4:** Comparison of water quality conditions.

Item	Weekly Water Quality Change			
	Adaptation Period		Challenge Period	
	1	2	3	4
pH	8.1	8.0	8.2	8.0
DO concentration	7.3	7.8	6.7	7.2
NH_3_-N concentration	0	0.25	0.25	0.25
NO_2_-N concentration	0.3	0.5	0.8	0.8

**Table 5:** Effect of *Lactobacillus plantarum* PSCPL13 on the blood profile of olive flounder.

Item	Control Group		Treated Group	
	Mean	SD	Mean	SD
WBC count (K/μL)	0.326	0.22	0.4486	0.34
NEU count (N%)	35.32	27.23	30.728	32.64
LYM count (L%)	30.28	17.97	49.758	33.6
MONO count (M%)	7.226	10.44	5.5576	6.36
EOS count (E%)	27.186	23.27	13.968	11.88
RBC count (K/μL)	3.586	0.49	3.644	0.18
HGB level (g/dL)	9.846	1.48	10.146	0.77
HCT (%)	61.72	8.854	62.08	3.03
MCV (fL)	172.2	4.15	170.4	3.65
MCH (pg)	27.46	1. 1	27.76	1.13
MCHC (g/dL)	15.92	0.33	16.3	0.53
RDW (%)	11.894	2.52	14.348	3.43
PLT count (K/μL)	33.74	16.36	25.6	12.07

## Data Availability

The data of this study will be available at reasonable request.
